# Microbial inoculants: reviewing the past, discussing the present and previewing an outstanding future for the use of beneficial bacteria in agriculture

**DOI:** 10.1186/s13568-019-0932-0

**Published:** 2019-12-21

**Authors:** Mariana Sanches Santos, Marco Antonio Nogueira, Mariangela Hungria

**Affiliations:** 1 Embrapa Soja, Cx. Postal 231, Londrina, Paraná 86001-970 Brazil; 2 Department of Biochemistry and Biotechnology, Universidade Estadual de Londrina, C.P. 60001, Londrina, Paraná 86051-990 Brazil

**Keywords:** Biological nitrogen fixation, Plant-growth-promoting bacteria, *Azospirillum*, PGPB, PGPR, Inoculation, Rhizobia, Chemical fertilizers

## Abstract

More than one hundred years have passed since the development of the first microbial inoculant for plants. Nowadays, the use of microbial inoculants in agriculture is spread worldwide for different crops and carrying different microorganisms. In the last decades, impressive progress has been achieved in the production, commercialization and use of inoculants. Nowadays, farmers are more receptive to the use of inoculants mainly because high-quality products and multi-purpose elite strains are available at the market, improving yields at low cost in comparison to chemical fertilizers. In the context of a more sustainable agriculture, microbial inoculants also help to mitigate environmental impacts caused by agrochemicals. Challenges rely on the production of microbial inoculants for a broader range of crops, and the expansion of the inoculated area worldwide, in addition to the search for innovative microbial solutions in areas subjected to increasing episodes of environmental stresses. In this review, we explore the world market for inoculants, showing which bacteria are prominent as inoculants in different countries, and we discuss the main research strategies that might contribute to improve the use of microbial inoculants in agriculture.

## Introduction

Humanity has always been concerned about food production to attend the increasing population and, for a long time, the solution was to expand agriculture to new areas. However, this scenario has changed in recent decades, first due to limitations of unexplored cultivable land, but also reinforced by the development of new technologies that allow higher yields, in addition to increasing environmental concerns, leading to agricultural practices aiming at achieving sustainable production. Therefore, although the global demand for food continues to increase, the concepts of agriculture sustainability, recovery of degraded areas, and mitigation of environmental impacts are gaining more respect (Canfield et al. 2010; Godfray et al. 2010). In this context, microbial inoculants—denominated as biofertilizers in some countries—have received increasing attention, gaining prominence and market scale in agriculture.

Inoculants are products that have in their composition living microorganisms capable of benefiting the development of different plant species. The most antique microorganisms used as inoculants are the “rhizobia”, diazotrophic bacteria able to colonize the rhizosphere and establish nodules in the roots of their host plants, composed by several species of the Fabaceae family. The symbiosis legumes-rhizobia leads to the process of biological nitrogen fixation (BNF), which very often can fully supply the plant´s demands on N. Moreover, other diazotrophic bacteria, such as *Azospirillum*, establish less straight relationships with the host plant, but are also able to supply, at least partially, the plant’s demands on N. Both *Azospirillum* and rhizobia, among other diazotrophic and non-diazotrophic bacteria are named as plant-growth-promoting bacteria (PGPB) or plant-growth-promoting rhizobacteria (PGPR), as they may benefit the plants by a variety of single or combined processes, including the production of phytohormones, siderophores, phosphate solubilization, induction of plant intrinsic systemic resistance to abiotic and biotic stresses, among others (Bhattacharyya and Jha 2012; Malusá and Vassilev 2014; Fukami et al. 2017, 2018a, b). Other microorganisms have also been increasingly used in agriculture for biological control of pests and diseases (Ciancio et al. 2016; Berg et al. 2017; Singh et al. 2017; Xiang et al. 2017), but this review will only deal with inoculants carrying strains that facilitate plant growth. Moreover, we will name all rhizobia and other bacteria carrying different mechanisms that promote plant growth as PGPB.

**Fig. 1:**
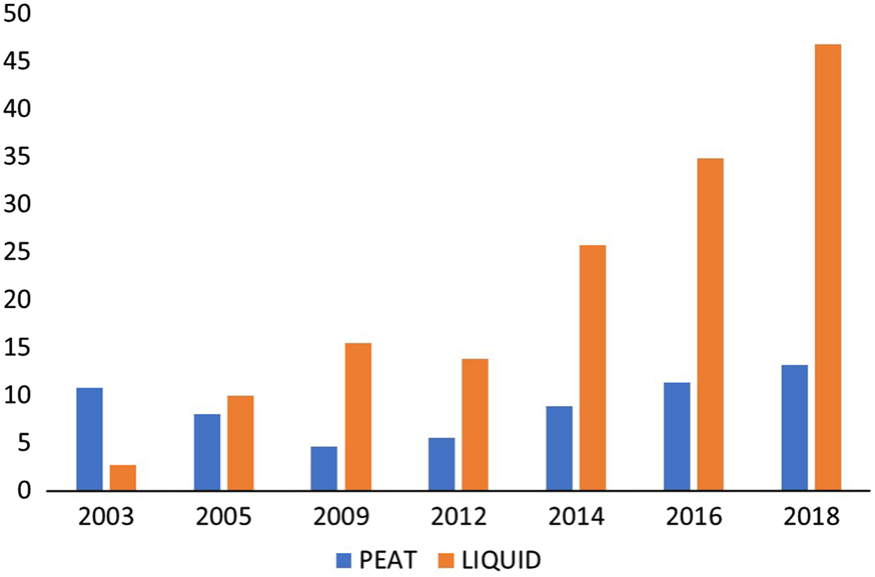
Market of microbial inoculants in Brazil in the last 15 years (million doses)

Currently, soybean (*Glycine max* (L.) Merr.) is the most inoculant-consuming crop worldwide, carrying bacteria belonging to the genus *Bradyrhizobium*. Brazil is probably the global leader in the use of inoculants for the soybean crop (Hungria and Mendes 2015; Okon et al. 2015; ANPII 2016) where approximately 78% of the copping area—nowadays 36.5 million hectares—is inoculated yearly (ANPII 2018). Additionally, inoculation of common beans (*Phaseolus vulgaris* L.), cowpea (*Vigna unguiculata* (L.) Walp.), maize (*Zea mays* L.) and co-inoculation of soybean and common bean with rhizobia and *Azospirillum* have also increased in Brazil (Hungria et al. 2010, 2015), so that the number of doses commercialized in the last years has impressively grown (Fig. 1). Other top countries in the use of inoculants are Argentina and India (Mazid and Khan 2014; Hungria and Mendes 2015; Okon et al. 2015; Sruthilaxmi and Babu 2017).

However, there are limiting factors that restrict the use of inoculants in some areas. Biotic and abiotic stresses may affect the effectiveness of the product, making them inefficient in cases such as nutrient-poor or unbalanced soils, salinity, water stress, increasing temperatures, pests and diseases, among others (Bashan et al. 2014; Das et al. 2017; Khan et al. 2017; Thilakarathna and Raizada 2017; Samago et al. 2018). To circumvent these factors, several studies have been addressed to gain better knowledge on the intrinsic properties of PGPB, seeking at understanding their optimum growth conditions and interaction with the host plants (Flores-Félix et al. 2018; Goulart-Machado et al. 2018; Jiménez-Gómez et al. 2018). Efforts have also been applied to improve the efficiency of microorganisms already available and in the identification of new elite strains to be used as inoculants under unfavorable and stressful environmental conditions, such as areas frequently experiencing drought, soils with low nutrient availability or with salinity, among others (Benidire et al. 2017; Koskey et al. 2017; Youseif et al. 2017). There is an increasing number of studies aiming to isolate, identify and evaluate the capacity of plant-growth promotion of bacteria with a variety of plant species, with potential to be transformed into new microbial inoculants in a near future (Yanni et al. 2016; Koskey et al. 2017; Manasa et al. 2017; Muleta et al. 2017).

Another technology with increasing application relies on the use of mixed inoculants, aiming to promote plant growth by combining distinct mechanisms of different microorganisms. Mixed inoculants can provide excellent results and show the great potential of being increasingly used by the farmers (Juge et al. 2012; Hungria et al. 2013, 2015; Chibeba et al. 2015; Bulegon et al. 2017; Ferri et al. 2017).

The objective of this short review is to explore the current market of inoculants, highlighting what has been produced and marketed lately in several countries, and the impact on agricultural sustainability. We also explore new ideas, new objectives and new strategies that are needed to generate information for the development of new products, breaking down barriers needed to expand the use of microbial inoculants in agriculture.

## Inoculant carriers

**Fig. 2:**
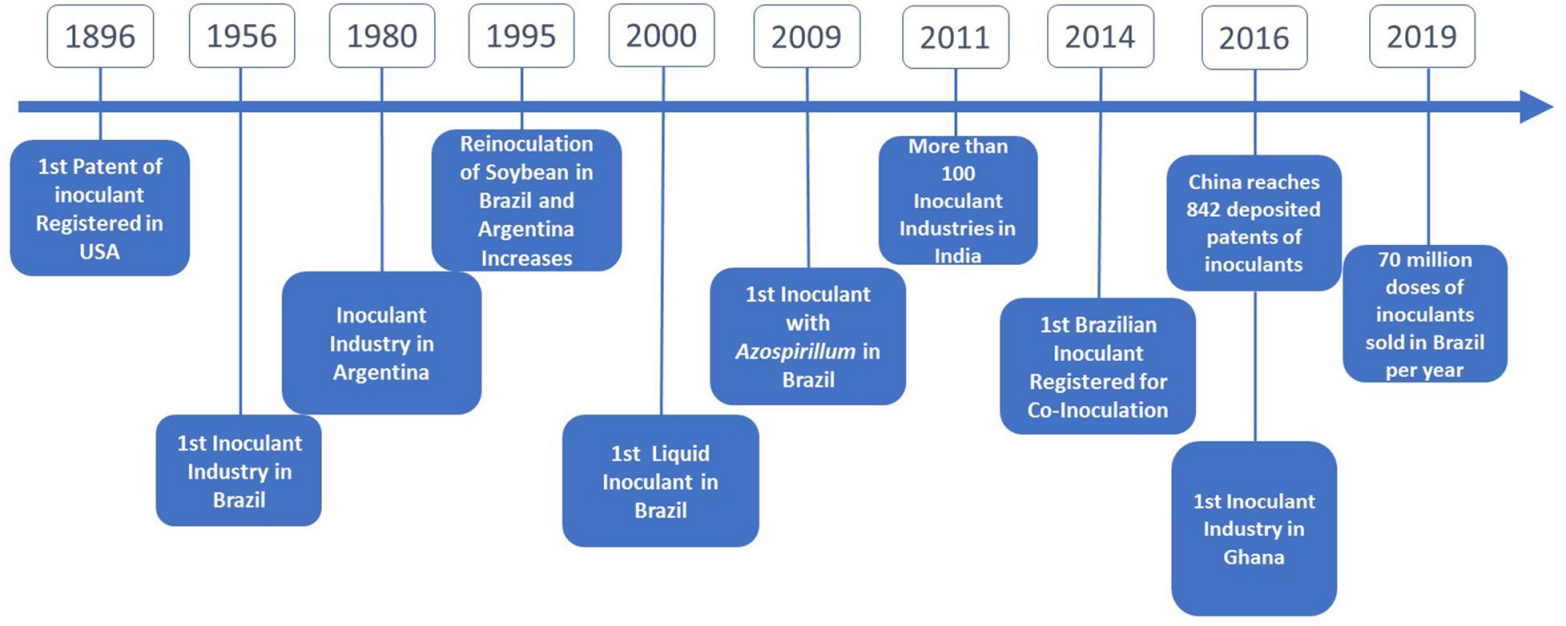
Chronology of some important steps in the development of microbial inoculants

Since the beginning of the manufacturing of inoculants, the industry has been concerned about generating increasingly efficient products, at a low cost, whose handling attends to the needs and the quality required by farmers. An important aspect is the choice of the carrier for the microorganisms, which should, among other things, provide long cellular viability and be of easy application. In 1896, in the USA, the first inoculant commercially produced, “Nitragin” (Fig. 2), used gelatin, and later, nutrient medium was employed as carrier for bacterial cells. Due to the high mortality rate, these carriers were soon replaced by peat, which remained as the “gold” carrier until the end of the 1990s, when the scenario began to change (Fig. 2) (Williams 1984).

Peat is a solid material, consisting of organic soil naturally occurring in specific environments and formed after a long geological period. The choice of peat as carrier for inoculants is due to its richness in organic matter, which serves as an important source of nutrients for the microorganisms. The peaty matrix also provides physical protection to the microorganisms against soil adversities and allows better cell survival in conditions of water restriction and high temperatures (Hungria et al. 2000a, 2005). In the process of seed inoculation with peat it is essential to use adhesives to help to stick the peaty matrix to the seeds; for example, in Brazil the most popular adhesive is 10% sucrose solution (Hungria et al. 2000a). The peat-based inoculant must be packed in sterilized polyethylene or polypropylene bags, with thickness of 0.06–0.38 mm, which preserves moisture but guarantees gas exchange with the external medium (Hungria et al. 2005).

Concerns about the use of peat as inoculant carrier rely on the exploitation of peat bogs, which may cause serious environmental impacts, including the destruction of habitats and CO_2_ emissions. In addition, in countries such as Brazil, where there are few peat bogs, importation of this material is required, increasing the production costs (Ribeiro et al. 2013). Due to these limitations, inoculants based on liquid formulations began to gain space, especially from the late 1990s onwards. In Brazil, the first liquid inoculant was approved by the Ministry of Agriculture for commercial used in 2000, and a decade later almost 80% of the inoculants sold in the country were in liquid formulations (Fig. 2); similar proportion is found in Argentina (ANPII 2018). Liquid inoculants consist of microbial cultures suspended in liquid medium rich in nutrients and cell protectors. They are easily handled and compatible with mechanized sowing, offering an advantage over solid inoculants at sowing. Another advantage is the easiness of sterilization, facilitating the absence of contaminants and, consequently, allowing higher cell concentration (Bashan et al. 2014; Cassán et al. 2015).

In addition to seed inoculation, liquid inoculants allow alternative application methods, such as in-furrow, and sprayed on soil or by “foliar” application (Campo et al. 2010; Fukami et al. 2016; Moretti et al. 2018). Alternative methods of application may be advantageous in some cases, for example, the inoculation in-furrow, to alleviate the impact of pesticides used for seed treatments in contact with the bacteria (Campo et al. 2010).

Other vehicles and methods for carrying microorganisms, such as agricultural and industrial residues, lyophilized bacteria and polymers for cell encapsulation, have been researched to develop more efficient and stable products. According to Bashan et al. (2014), industrial residues and agricultural by-products such as sugarcane bagasse, sawdust or brewery waste can be used as carriers for inoculation of microorganisms. However, the major limitation for the use of these raw materials is their poorly consistent composition, and often difficulties for sterilization.

As one of the challenges for inoculant production is to maintain cell viability for long period, lyophilization and freezing of microbial cells have emerged as possibilities to overcome this problem. The process of lyophilization consists of removing the intracellular water, reducing the metabolic activity and increasing microbial lifetime. The dry cell culture must be mixed with a liquid or gel formulation at sowing. The great barrier to the commercial production of inoculants with lyophilized microorganisms is the high production cost because it requires specialized equipment and skilled labor (Williams 1984; Hungria et al. 2005). Besides, the time and conditions needed for cell recovering in liquid or gel formulations represent barriers for the adoption of inoculants by farmers, especially high large areas are cropped, such as for the soybean crop in South America.

The encapsulation of living microbial cells with polymers, such as alginate and polyacrylamide has also been increasingly mentioned. For the encapsulation, the liquid inoculant containing bacterial culture is mixed with an adjuvant polymer, capable of causing solidification. The most used method consists of mixing dropwise the microbial culture in a solution containing calcium chloride, resulting in solid beads with high cell concentration. The spheres are placed in contact with the seeds at sowing time and the bacteria are slowly released. These spheres are biodegradable and do not cause environmental impact. Encapsulation confers protection to the cells for high temperature and environmental stresses and are also easy to handle. Once again, the economic factors have always represented the main obstacle for scaling the industrial production (Bashan 1986; Bashan et al. 2002; Date 2001).

Great efforts have been applied by several industries to develop new products able to attend the new requirements of the market and compatible with new technologies. The trend for this next decade is to apply considerable investment in innovation, searching for new inoculant formulations to hit the ever growing market.

## Inoculants containing mixes of bacteria

The great majority of the first manufactured inoculants contained only one species of microorganism, and in general one strain, the one with the best inoculation results for a particular crop. Exceptions included a maximum of two microorganisms “of the same type”, for example, two *Bradyrhizobium* strains or species for soybean. The use of two strains in the same inoculant would increase the chances that at least one would nodulate and perform well with the legume. For example, in Brazil, the combination of two *Bradyrhizobium* strains for the soybean crop has been preferentially used by the farmers since the 1950s (Hungria et al. 1994; Hungria and Mendes 2015).

Particularly in the last decade, the use of inoculants containing microorganisms of “different type” has expanded. The idea is of combining strains or species acting in different microbial processes, so that the combined benefits of each one would result in higher benefits and, ultimately, yields. Examples of mixed inoculant are those combining microorganisms whose major processes are BNF (e.g. *Bradyrhizobium* spp., *Rhizobium* spp.) and phytohormone production (e.g. *Azospirillum* spp., *Pseudomonas* spp.), solubilization of phosphate (e.g. *Bacillus* spp.), or biological control (e.g. *Pseudomonas* spp., *Bacillus* spp.). If the microorganisms cannot be combined in a single product, they are manufactured separately and the bags containing each one are sold in the same package.

The application of mixed inoculants is usually called co-inoculation or mixed inoculation and it is currently possible to find co-inoculants for several crops in the market. The efficiency of co-inoculation is closely related to the appropriate selection of strains, the cellular concentration of each one, method of inoculation (applied to the seeds, leaf-spray, in-furrow), and to the plant genotype. Therefore, research is needed to generate knowledge aiming at the production of new formulations for commercial inoculants with mixed bacteria (Cassán et al. 2015), and on alternative methods of application of inoculants and microbial molecules (Campo et al. 2010; Fukami et al. 2016).

In Brazil, co-inoculation of *A. brasilense* with *Bradyrhizobium* spp. for the soybean crop and with *Rhizobium tropici* for the common beans was launched in 2014 and impressive increases in grain yield have been reported (Hungria et al. 2013, 2015; Souza and Ferreira 2017; Nogueira et al. 2018). Even in areas with high population of compatible rhizobia for both crops (> 10^4^ cells of compatible rhizobia/g soil), for the soybean crop single inoculation of *Bradyrhizobium* resulted in mean increases of 8.4% in grain yield compared with the naturalized population, whereas the co-inoculation with *A. brasilense* promoted an “upgrade” to 16.1%; for common beans, single inoculation with *R. tropici* increased yield by 8.3%, whereas the co-inoculation improved the yield by 19.6% (Hungria et al. 2013) (Table 1). Since them, other benefits attributed to the co-inoculation of soybean with *Bradyrhizobium* and *Azospirillum* in Brazil are the promotion of early nodulation (Chibeba et al. 2015), and increased tolerance to moderate water restriction (Cerezini et al. 2016; Silva et al. 2019).

**Table 1:** Examples of studies comprising inoculation of various plant species with specific bacterial strains resulting in increased grain yield

Crop	Microorganism	Strains	Increase in grain yield compared with the non-inoculated control (%)	References
Soybean	*Bradyrhizobium japonicum*	–	4.5	Hungria et al. (2001)
	*B. japonicum*	SEMIA 5079 and SEMIA 5080	8.4	Hungria et al. (2013)
	*B. japonicum*	532 C and USDA 110	12–19	Ulzen et al. (2016)
	*B. japonicum*	–	1.6–6.3	Leggett et al. (2017)
Common beans	*Rhizobium tropici*	SEMIA 4080 (= PRF 81)	31.6–36	Hungria et al. (2000b)
	*R. tropici*	SEMIA 4080	8.3	Hungria et al. (2013)
	*R. tropici*	CPAO 12.5 L2	66	Mercante et al. (2017)
	*Rhizobium leguminosarum* sv. phaseoli	HB-429	48	Samago et al. (2018)
Cowpea	*B. japonicum*	BR 3267	38.1	Ulzen et al. (2016)
	*Bradyrhizobium liaoningense*	VIBA-1	54.8	Padilla et al. (2016)
	*Bradyrhizobium yuanmingense*	VIBA-2	38.3	Padilla et al. (2016)
Faba beans	*R. leguminosarum* sv. *viciae*	NGB-FR 126	46.8–81.4	Youseif et al. (2017)
	*R. leguminosarum* sv*. vicieae*	NSFBR-30 and HUFBR-15	5–75	Argawa and Mnalku (2017)
Maize	*Azospirillum brasilense*	Ab-V5 and Ab-V6	27	Hungria et al. (2010)
	*A. brasilense*	Ab-V5	29	Ferreira et al. (2013)
	*A. brasilense*	Ab-V5 and Ab-V6	14.3	Galindo et al. (2019)
	*Pseudomonas fluorescens*	–	29–31	Sandini et al. (2019)
Wheat	*Bacillus polymyxa*	Bp 4317	13.6–19.5	Rodriguez-Caceres et al. (1996b)
	*A. brasilense*	Sp246	14.7	Ozturk et al. (2003)
	*A. brasilense*	Ab-V5 and Ab-V6	31	Hungria et al. (2010)
	*A. brasilense*	–	18	Karimi et al. (2018)
Rice	*Burkholderia vietnamiensis*	TVV75	22	Tran et al. (2000)
	*B. vietnamiensis*	MGK3	12.1	Govindarajan et al. (2007)
Tomato	*A. brasilense*	Sp-7	11	Alfonso et al. (2005)
	*P. fluorescens*	SS5	57	Ahirwar et al. (2015)
Co-inoculation				
Soybean	*A. brasilense* and *B. japonicum*	Ab-V5 and Ab-V6; SEMIA 5079 and SEMIA 5080	14.1	Hungria et al. (2013)
	A. brasilense* and B. japonicum*	Ab-V5 and Ab-V6; SEMIA 5019 and SEMIA 5079	81.9	Ferri et al. (2017)
Common beans	A. brasilense* and *R. tropici*	Ab-V5 and Ab-V6; SEMIA 4080	19.6	Hungria et al. (2013)
Wheat	*Serratia marcescens*, *Microbacterium arborescens*, and *Enterobacter* sp.	–	24	Kumar et al. (2017)
Rice	*Klebsiella pneumoniae*, *P. fluorescens*, and *Citrobacter freundii*	4P, 1N and 3C	17.5	Nguyen et al. (2003)
	*P. fluorescens*, *Bacillus subtilis*, *Bacillus amyloliquafaciens* and *Candida tropicalis*	1N, B9, E19 and HY	26.7	Nguyen (2008)
	*A. brasilense* and *P. fluorescens*	–	20.2	de Salamone et al. (2012)

All experiments were carried out under field conditions with seed inoculation, except those marked (*), which inoculation occurred in-furrow. Yield increase varied between studies because of specific cropping conditions such as soil composition, temperature, site and environmental conditions

In addition to *Azospirillum* spp., several other PGPB have been reported as successful in co-inoculation trials with soybean, as *Pseudomonas* sp. (Egamberdieva et al. 2017; Pawar et al. 2018), *Actinomyces* sp. (Nimnoi et al. 2014), *Bacillus* sp. (Atieno et al. 2012; Subramanian et al. 2014; Petkar et al. 2018). Improvements in yields have also been reported with the co-inoculation of rhizobia presenting different mechanisms of action. For example, Jesus et al. (2018) verified benefits by the co-inoculation of common bean with *R. tropici* CIAT 899, *Bradyrhizobium diazoefficiens* USDA 110 and *Bradyrhizobium elkanii* 29w; according to the authors, *Bradyrhizobium* spp. would improve the symbiosis efficiency of *Rhizobium*, resulting in greater number of nodules, biomass production and N accumulation. The suggested mechanism is that *Bradyrhizobium* sp. co-inoculated produces signaling molecules, such as nodulation factors (Nod factors) and surface polysaccharides that stimulate root nodulation by *R. tropici*, improving the efficiency of BNF.

Co-inoculation has also been shown to be efficient under several limiting conditions, such as in low phosphate soils. Generally, the BNF is compromised under these situations, but the co-inoculation with phosphate-solubilizing microorganisms can make it available for plant nutrition and, in the case of legumes, help to ensure the benefits of BNF (Jorquera et al. 2008; Morel et al. 2012; Shiri-Janagard et al. 2012; Korir et al. 2017). For example, Korir et al. (2017) evaluated the effects of co-inoculation in common beans grown in a soil with low P and observed that plants inoculated with *Rhizobium* strain IITA-PAU 987 and *Bacillus megaterium* increased nodulation, shoot dry weight and had 31% increase in BNF when compared with the single inoculation with *Rhizobium*.

## Main inoculated crops

### Soybean

Soybean is an annual herbaceous dicotyledonous, originally grown in the eastern region of Asia (Aliyev and Mirzoyev 2010). Until the nineteenth century, its cultivation remained restricted to the eastern countries, and spread to other continents, as America and Africa, only at the end of this period (Dall´Agnol et al. 2007; Aliyev and Mirzoyev 2010). Nowadays, the main soybean producers are the USA, Brazil, and Argentina.

Soybean is probably the most successful example of crop benefiting from the application of microbial inoculants, more specifically, carrying *Bradyrhizobium* spp. strains. South American countries lead soybean inoculation. In contrast, in the USA, estimates are that only 15% of the area cropped with soybean has been inoculated, what might be related to the low cost of N-fertilizer marketed in the country (Chang et al. 2015). The low cost of N-fertilizer may also have implied in lower interest in innovation of technologies updated with new agricultural practices.

The Brazilian research for the production and commercialization of inoculants is very advanced and the country has one of the most complete legislation in this area. Common resolutions for inoculants commercialization were defined in 1998 for the Mercosur, the common market including Brazil, Argentina, Uruguay and Paraguay. Following, in Brazil, a legislation of 2004 included definitions and norms on specifications, guarantees, registrations, packaging and labeling of inoculants, as well as the list of the microorganisms that could be used in commercial inoculants in the country; the document was updated in 2011 (MAPA 2004, 2011). Nowadays, four strains of *Bradyrhizobium* are authorized for the production of soybean inoculants in the country (*Bradyrhizobium japonicum* SEMIA 5079 (= CPAC 15), *B. diazoefficiens* SEMIA 5080 (= CPAC 7), *B. elkanii* SEMIA 5019 (= 29w) and SEMIA 587). The legislation still establishes a minimum concentration of viable cells (1 × 10^9^ viable cells/g or mL) of the inoculant until the expiration date, which must be at least 6 months, and void of contaminants at the 1 × 10^−5^ dilution (Hungria et al. 2010; MAPA 2011). The technical recommendation in Brazil indicates a dose that allows at least 1.2 million viable cells/seed to guarantee a successful nodulation (Hungria et al. 2017; Hungria and Nogueira 2019). The credibility of the inoculant market in Brazil relies on strict legal regulation. Interestingly, the legislation was created based mainly on the Australian legislation, where nowadays the regulation relies on an agreement between partners, as a voluntary control (Bullard et al. 2005; AIRG 2010).

In Brazil, the inoculation of soybean with elite *Bradyrhizobium* spp. strains can fully supply the crop’s demand on N, dismissing the use of N-fertilizers. Probably as a result of breeding for BNF, the symbiosis with soybean is very sensitive to N-fertilizers, drastically reducing nodulation (Hungria et al. 2007; Hungria and Mendes 2015). Soybean cropping without any N-fertilizer has generated an annual economy that today is estimated at about 20 billion dollars.

In Brazil, Argentina and in other South American countries, successful results have been achieved with the re-inoculation of soybean, i.e., the yearly inoculation even in soils presenting well-established compatible rhizobial population from previous inoculations (Hungria et al. 2001; Hungria and Mendes 2015). This practice led to the commercialization of over 70 million doses of inoculants for soybean in Brazil in the last crop season. Estimates in Brazil are that re-inoculation increases soybean grain yield by 8% in average (Hungria and Mendes 2015) and by 6.8% (Leggett et al. 2017) to 14% (Hungria et al. 2016) in Argentina. In the USA, re-inoculation is traditionally not recommended, based on results from a former study showing that rhizobial populations as low as 10 cells/g would inhibit the nodule formation by inoculant strains (Thies et al. 1991a, 1995). However, mean yield increases due to inoculation considering areas of traditional soybean cropping have been recently estimated at 1.67% (Leggett et al. 2017), but could probably be higher if high N-fertilizer levels were not applied to the crops comprising the soybean agricultural systems (Chang et al. 2015). Amazingly, even the most recent studies on the quantification of soybean BNF in the USA take into consideration a large number of sites, soil fertility, and application of mineral N, but not the re-inoculation component (Córdova et al. 2019). Certainly, the annual re-inoculation is responsible for the high contribution of BNF to the soybean N nutrition in Brazil, with values as high as 94% of the aboveground N accumulation (Hungria et al. 2006), while in the USA these values range from 23 to 65% (Córdova et al. 2019).

The Sub-Saharan Africa (SSA) region has developed, over the years, strategies for the use of beneficial microorganisms in soybean adapted to local environment and social characteristics. As consequence of the lack of local production and difficulties in the importation of inoculants in the 1970s, the International Institute of Tropical Agriculture (IITA) launched a breeding program aiming at developing high-yielding tropical soybean varieties capable of nodulating with indigenous rhizobial strains. These new varieties were named “TGx” (tropical *Glycine* cross) or “promiscuous” soybeans (Kueneman et al. 1984; Pulver et al. 1985), and contributed to the expansion of soybean production in the SSA.

Because the usually acidic, saline, and low organic matter of the SSA soils, the average soybean yield is usually well below the world average (Thuita et al. 2012; Muleta et al. 2017). Therefore, in addition to the soybean genetic breeding, further studies have been carried out aiming at increasing yields. For example, in Ethiopia, Muleta et al. (2017) searched for acid-tolerant rhizobia as strategy to increase soybean performance. A local isolate was able to improve soybean yield, indicating that search for indigenous or naturalized elite isolates might represent an interesting strategy to be adopted in other African countries. Impressive yield increases have also been observed by combining application of P-fertilizer and rhizobial inoculant in Nigeria (Ronner et al. 2016), and along with other studies suggest that P is probably the main limiting factor to the BNF in Africa (Vanlauwe et al. 2019).

In Mozambique, the majority of soybean cropping was with promiscuous varieties without inoculation; however, due to the increased demand on exportation of grains and poultry industry, the cultivation of non-promiscuous and more-productive cultivars associated with inoculation has increased (Dias and Amane 2011). As the agroclimatic conditions of the soybean production areas in Mozambique are similar to the main areas of soybean cultivation in the Brazilian savanna, Chibeba et al. (2018) evaluated and confirmed that elite strains identified in Brazil could have a successful performance in Mozambique with non-promiscuous soybean genotypes. The feasibility of transferring inoculation technologies between countries is of outstanding importance, as it can accelerate the establishment of sustainable cropping systems, saving time, labor and money. However, it is always desirable to search for indigenous or adapted strains, and promising local soybean strains have been identified in Mozambique (Chibeba et al. 2017), in a near future, their performance should be compared with the imported strains under field conditions.

### Common beans

Similar to soybeans, common beans (*Phaseolus vulgaris* L.) are cropped worldwide, representing the most important source of protein in several countries, especially in South and Central America and Africa (Hungria et al. 2000b, 2013; Ribeiro et al. 2013). Although Brazil is one of the main producers (3.17 million hectares in the 2017/2018 crop season) and consumer of common beans worldwide, grain yields are usually low, bellow 1000 kg/ha (Hungria et al. 2007; CONAB 2019). Therefore, many strategies have been considered to improve yield, concomitantly to the tolerance to environmental stresses, at low cost.

Studies carried out in Brazil identified two strains of the “*R. tropici* group” for common bean that show high BNF rates, competitiveness, tolerance to environmental stresses and genetic stability (Hungria et al. 2000a, 2003; Mostasso et al. 2002). The strains PRF 81 (= SEMIA 4080) of *R. freirei* and H 12 (= SEMIA 4088) of *R. tropici* have been used in commercial inoculants in Brazil since 1998 and 2004, respectively, in addition to *R. tropici* CIAT 899, originally isolated in Colombia by Dr. Peter H. Graham (Hungria et al. 2000a, 2003; Gomes et al. 2015). Interestingly, CIAT 899 has been recognized as an outstanding strain in several countries (Gomes et al. 2015; Vanlauwe et al. 2019).

The use of inoculants for common bean favors yields, but there are reports indicating that BNF might not replace N-fertilizers completely, especially in soils where the N concentration is very low. Studies suggest that the application of 15 or 20 kg N/ha along with inoculation at sowing might improve grain yield (Soares et al. 2016), but higher doses of N at sowing may lead to reduced nodulation (Hungria et al. 2003). Noteworthy, Mercante et al. (2017), in a series of field trials performed in the Brazilian Cerrados verified that, in comparison with the indigenous population, the mean increase in grain yield by inoculating *R. tropici* CIAT 899 was of 410 kg/ha, but decreased to 365 kg/ha with the application of 20 kg of N/ha at sowing; a new identified elite strain resulted in outstanding mean increases of 665 kg/ha in grain production (Table 1).

The African continent also stands out in the production and consumption of common beans. Estimates are that 25% of the total world area cropped with common beans are in Africa, where the legume is part of the diet of more than 100 million people (Aserse et al. 2012; Beebe et al. 2013), with Tanzania, Kenya, Uganda and South Africa been the main producers (USDA 2012). Similar to South America, African researchers are studying different ways of increasing common bean yield by using BNF, especially in situations where the efficiency of nodulation by *Rhizobium* is compromised, such as dry conditions, low P concentrations, soil salinity and high temperatures (Yanni et al. 2016; Samago et al. 2018). In order to identify rhizobia capable of tolerating drought and salinity stresses, Yanni et al. (2016) selected indigenous strains in the eastern and western regions of the Nile delta, and identified elite strains with good performance under saline and water stress conditions, promising for the use as inoculants (Kanonge-Mafaune et al. 2018).

The approach of selecting adapted indigenous strains with high capacity of BNF was also investigated by Koskey et al. (2017) in soils of low fertility in Kenya. Regarding the symbiotic efficiency, four indigenous isolates showed good symbiotic performance, one being able to increase grain yield by 30% in comparison to the commercial inoculum for beans, Biofix (strains not informed). The importance of P for the symbiotic performance of common bean was highlighted under field conditions in Nigeria (Ronner et al. 2016) and Ethiopia (Samago et al. 2018).

### Cowpea

Originated from the African continent, cowpea (*Vigna unguiculata* L. Walp.) is the major legume cropped in many African countries, responsible for more than 95% of the world’s production (Silva et al. 2016). In Brazil, cowpea was introduced in the sixteenth century and has been cultivated mainly in the North and Northeast regions. Despite the still modest yield, Brazil has exported cowpea grains to some countries such as India, Egypt and Pakistan (Silva et al. 2016).

Cowpea is usually tolerant to high temperatures, low soil fertility and water restriction; grain yield can be limited by N availability, which can be supplied by BNF. Interestingly, African countries with climate and humidity conditions similar to the North and Northeast of Brazil have tested and observed positive responses to inoculation with elite *Bradyrhizobium* strains from Brazil. Boddey et al. (2016) and Ulzen et al. (2016) observed significant increases in nodulation and yield of cowpea inoculated with Brazilian rhizobia in northern Mozambique and northern Ghana.

Other indigenous microorganisms have also been identified, selected and proved to increase cowpea yield. A study carried out in a saline soil in Cuba demonstrated the efficacy of two indigenous strains (*Bradyrhizobium liaoningense* VIBA-1 and *Bradyrhizobium yuanmingense* VIBA-2) (Padilla et al. 2016) (Table 1). In another study, in Bangladesh, one strain isolated from cowpea nodules was identified as *Rhizobium* sp. SOY7 and presented excellent results of nodulation and plant growth, when compared with the non-inoculated control (Nushair et al. 2017).

### Faba beans

Used in Chinese cooking for at least 5000 years, the origin of faba beans (*Vicia faba* L.) is still controversial (Duc 1997). Currently, the crop is produced and consumed in several countries, due to its adaptation to various climatic zones. The main producers are China, Italy, Spain, United Kingdom, Egypt, Ethiopia, Morocco, Russia, Mexico and Brazil (Duc et al. 2010; Lavania et al. 2015). However, there has been a considerable decline in the cropped area worldwide, mainly due to susceptibility to environmental stresses, affecting yield stability (Rubiales 2010).

In relation to the capacity of BNF, many soils favor the development of compatible rhizobial strains (Köpke and Nemecek 2010). The identification of rhizobia from nodules of faba beans indicate that the most common species are *Rhizobium leguminosarum* bv. viciae, *Rhizobium fabae*, *Rhizobium laguerreae* and *Rhizobium anhuiense* (Mutch and Young 2004; Tian et al. 2008; Saïdi et al. 2014; Zhang et al. 2015). Because of the high population of rhizobia in areas cropped with the legume for a long time, inoculation is usually not adopted. However, in regions where faba beans are not intensively cropped, or under stressful conditions, inoculation can benefit plant development (Köpke and Nemecek 2010; Youseif et al. 2017).

Faba beans are one of the most consumed grains in Egypt. Despite the predominantly low-fertility soils, inoculation is usually not performed and grain production is low, not attending the country’s demand. However, the potential of response to inoculation has been demonstrated in some studies, e.g. Youseif et al. (2017) evaluated 17 indigenous rhizobial strains from different regions of Egypt, and observed that seed inoculation increased grain yield (Table 1) and N accumulation, reaching up to 155 kg ha of N in grains.

In saline soils in Morocco, Benidire et al. (2017) reported two indigenous strains of *R. leguminosarum* (RhOF34 and RhOF125) that induced plant protection against salinity, leading to increases in nodulation, plant biomass and N content, confirming that indigenous species may have excellent results when inoculated in fava beans.

### Other legumes

Legumes are generally part of the food base of people and animals throughout the world. In addition to soybeans and various types of beans, other crops are also important sources of protein and nutrients and serve as raw materials for many industrialized products. Therefore, raising the yield of these crops under a variety of environments, by means of inoculation with elite rhizobial strains has been the subject of several studies in several countries.

In Brazil, *Bradyrhizobium* sp. strain SEMIA 6144, originally from Africa, has been used in commercial inoculants for peanut (*Arachis hypogaea*); however, inoculation is not a common practice for this crop in the country, attributed to the lack of response, due to the high population of indigenous rhizobia. Indeed, peanut is a very promiscuous species capable of nodulating with a broad range of soil rhizobia (Thies et al. 1991b). However, an efficient contribution of the BNF may require specific elite strains, adapted to local biotic and abiotic conditions and may vary with the plant genotype. For example, Marcondes et al. (2010) evaluated the BNF efficiency of isolates from two peanut varieties (IAC 886 Runner and IAC Tatu ST) and verified that the bacteria performance varied with the plant genotype.

In 2017 the first inoculant was produced for peanuts in Africa, 1 year after the establishment of the first industrial plant for inoculant production in Ghana, in a partnership with Brazil (Fig. 2). Although it is still in the testing phase, the results are promising and peanut growers are expected to benefit from inoculation in the coming years.

Chickpea (*Cicer arietinum*) is a highly nutritive legume cropped mainly in India, but also in more than fifty other countries (Jukanti et al. 2012). Bacteria of the genus *Mesorhizobium* sp. are commonly found in association with chickpea (Laranjo et al. 2014) and *Mesorhizobium ciceri* has already been indicated for the production of inoculants. In Australia, *M. ciceri* strain CC1192 has been used in inoculants since the 1970s (Bullard et al. 2005). Besides, several studies have been carried out to identify indigenous strains capable of nodulating and promoting chickpea growth, even in unfavorable environments, such as low-fertility soils (Tena et al. 2016; Pandey et al. 2018).

Guar (*Cyamopsis tetragonolobus* L.) is a legume that has gained prominence in global agriculture due to several industrial uses, as their seeds are rich in galactomannan gum, which can be used as lubricant, binder, thickener and emulsifier. It is cultivated in several semi-arid regions such as in India, Pakistan and the United States (Ibrahim et al. 2016; Thapa et al. 2018). Similar to other legumes, guar has the potential to associate with rhizobia, but the process of nodulation with rhizobia is still not well known (Abidi et al. 2015); therefore, studies have been performed to identify elite rhizobial strains (Ibrahim et al. 2016; Khandelwal and Sindhu 2012). Thapa et al. (2018) evaluated two guar varieties inoculated with two rhizobial inoculants, one composed by a complex mixture of *Rhizobium* and the other carrying *Rhizobium* USDA 3385, on two soils of different textures, and promising results were found, as abundant nodulation, incentivizing further experiments.

An increasing number of yields increase have been reported for important crops such as soybeans, common beans and chickpeas inoculated with elite rhizobial strains, leading to interest in using microbial inoculants for several other legumes. However, it has also increased the interest for the use of other plant-growth promoting bacteria in non-legumes.

### Maize

Maize (*Zea mays* L.) is a native grass from Central America (Doebley 1990a, b), and currently the third most cultivated cereal in the world. The interest in maize production is due to its versatility and broad use, ranging from human and animal feed to the production of biofuel, and also as an input in the manufacture of many products (Awika 2011). The main producers and consumers are the USA and China, followed by Brazil (DERAL 2019).

Maize can associate with PGPB, particularly those belonging to the genus *Azospirillum*, which are currently used as inoculants for this crop worldwide. Mexico was one of the first countries to commercialize inoculants for maize carrying *Azospirillum* in 2002 (Reis 2007), followed by Argentina.

Brazil has a long tradition in studies with *Azospirillum*, carried initially by Dr. Johanna Döbereiner. She described the capacity of *Azospirillum*, originally named as *Spirillum*, to perform BNF when associated with grasses. In 1978 the species *Spirillum lipoferum*, initially described by Beijerinck (1925), was reclassified as *Azospirillum*, with the prefix “azo” added as a reference to the term “azote,” nomenclature given by Lavoisier to nitrogen. At that time, the genus comprised two species, *Azospirillum lipoferum* and *Azospirillum brasilense* (Tarrand et al. 1978). Other species of *Azospirillum* were described in the following years, so that in 2019 the genus comprises 21 species (DSMZ 2019).

However, it was only in 2009 that the first commercial strains of *A. brasilense*, Ab-V5 and Ab-V6, were released for the use in commercial inoculants for maize and wheat (*Triticum aestivum* L.) in Brazil (Hungria et al. 2010; MAPA 2011). In maize, these strains resulted in increases in grain yield that reached 27%, compared with the non-inoculated control (Hungria et al. 2010) (Table 1). Since the release of the first commercial inoculant for grasses in Brazil, in 2009 (Fig. 2), the number of sold doses of inoculants carrying *A. brasilense* has grown significantly, reaching about 7 million doses in the 2017/18 crop season. In Argentina, the market of *Azospirillum* has started before Brazil, with the commercial strain *A. brasilense* Az39 selected in the 1980s and able to increase maize and wheat yields from 13 to 33% (Cassán et al. 2015; Cassán and Diaz-Zorita 2016).

In addition to its ability for BNF, numerous studies have demonstrated other properties of *Azospirillum*, the most important being the capacity for synthesizing phytohormones. Many of these molecules are related to root development, positively influencing their growth, resulting in greater absorption of nutrients and water from soil (Bashan and De-Bashan 2010; Ardakani and Mafakheri 2011; Fukami et al. 2017, 2018a, b). Therefore, grasses associated with *Azospirillum* present root structure capable of absorbing larger amounts of nutrients and water (Bashan and De-Bashan 2010). Auxins (Fallik et al. 1989; Fukami et al. 2017), gibberellins (Janzen et al. 1992; Cohen et al. 2009), ethylene (Perrig et al. 2007), cytokinins (Strzelczyk et al. 1994; Abbasi et al. 2015) and salicylic acid (Perrig et al. 2007; Cohen et al. 2009; Fukami et al. 2017) are the most commonly cited molecules.

Turan et al. (2012) emphasized the capacity of P solubilization by some strains of *Azospirillum*, increasing P availability in the soil and yields of wheat. Some strains of *Azospirillum* may also attenuate damages caused by abiotic stress, such as salinity and drought, as well as biotic stresses, like plant resistance against pathogens (Bashan and De-Bashan 2010; Fukami et al. 2018a).

Despite the benefits of *Azospirillum* in cereals, the bacterium is not able to supply all N demand, requiring the application of complementary doses of N. However, the amount of N-fertilizer to achieve high yields can be reduced by 25 to 50% (Hungria et al. 2010; Piccinin et al. 2013; Fukami et al. 2016).

Although *Azospirillum* is mainly inoculated on the seeds due to easiness and low doses (Cassán et al. 2015), the seed treatment with pesticides is potentially harmful and may impair the survival and metabolism of the inoculated cells. To overcome such problem, alternative methods of inoculation via foliar, in-furrow or soil spraying can be used. Fukami et al. (2016) evaluated the responses of maize inoculated with *Azospirillum* in-furrow, via soil spraying at sowing or via leaf spraying after seedlings had emerged, in comparison seed inoculation. Positive results were obtained with both alternative methods of inoculation, but higher doses were required than inoculation via seeds.

Besides *Azospirillum*, other groups of PGPB have been studied in inoculation of maize, such as *Pseudomonas* spp. (Burr et al. 1978; Ahirwar et al. 2015; Thirumal et al. 2017; Sandini et al. 2019). *Pseudomonas* are able to produce siderophores, which are molecules capable of capturing insoluble iron from the environment (Fe^3+^), and convert it to a soluble form (Fe^2+^) available for plants (Sharma and Johri 2003; Sah et al. 2017). Considering that iron is essential for metabolism and consequently, for plant development, the siderophores-producing microorganisms can positively improve plant development in Fe-deficient environments.

The production of siderophores by *P. aeruginosa* strains RSP5 and RSP8 was demonstrated in iron sufficient and iron-deficient soil (Sah et al. 2017). The strain RSP5 produced more siderophores in both soils and improved the Fe uptake by maize, in addition to increases in shoot and root length, number of spikes and number of grains. However, we must emphasize that many PGPB may also be highly pathogenic to humans, animals and plants. Therefore, it is critical to evaluate the non-pathogenicity of the strains before thinking about any use as inoculant and, certainly, *P. aeruginosa* is not a proper candidate for a commercial inoculant.

The use of *Bacillus* strains as inoculants is also increasing, in replacement to fertilizers. In Brazil, strains have been selected that improve P mobilization, by mechanisms as phytohormones production and P solubilization, this last one attribute to acid production by the bacteria (de Abreu et al. 2017). In Brazil, elite strains of *Bacillus* proved to improve P uptake production of grasses (Ribeiro et al. 2018), and the first commercial inoculant carrying P-solubilizing bacteria (*Bacillus subtilis* and *B. megaterium*) was released in 2019, with great acceptance by the farmers.

### Wheat

Wheat is a cereal of global importance for human and animal feeding and can also benefit from inoculation with *A. brasilense* (Bashan et al. 2004; Hungria et al. 2010). In the 1980s an important study was carried out in Mexico on the inoculation of wheat with *Azospirillum*. The concentration of the inoculant was 3–5 × 10^8^ CFU/g and the dose applied of 15 g/kg seed. Inoculation caused significant increases in yield, from 23 to 63% in 1986, and from 24 to 43% in 1987. The best results were obtained with strain Cd and with a local *A. brasilense* strain isolated from the rhizosphere of *Brachiaria mutica* (UAP-55) (Caballero-Mellado et al. 1992).

In the following decade, in Argentina, many studies were carried out with inoculation of *Azospirillum*. In 1992–1993 two experiments were carried out with inoculation of strains Az39 and Cd on wheat under greenhouse conditions using soil from a semiarid region of Argentina. Az39 and Cd strains increased the grain yield by 30% and 16%, respectively, and both increased the root dry weight compared with the non-inoculated control (Rodriguez-Caceres et al. 1996a). Nowadays, Az39 is the major strain used in commercial inoculants in Argentina (Okon et al. 2015).

In Brazil, Hungria et al. (2010) observed 13 to 18% increase in grain yield of wheat inoculated with *A. brasilense* Ab-V1, Ab-V5, Ab-V6 and Ab-V8 strains. When the strains Ab-V5 and Ab-V6 were combined, wheat yields increased by 31%; therefore, inoculant industries have mixed both strains in wheat inoculants (Hungria et al. 2010) (Table 1).

Further beneficial action of *A. brasilense* has been reported on wheat, such as the photo-protection of photosynthetic pigments and increase of proton efflux of roots, positively affecting plant development (Bashan et al. 1989, 2005).

Successful wheat inoculation with *Azospirillum* has also being reported in Israel (Kapulnik et al. 1983, 1985), England (Harris et al. 1989), Egypt (El-Lattief 2012), and Pakistan (Zaheer et al. 2019). Unfortunately, despite numerous studies proving the benefits of wheat inoculation, this practice is poorly adopted, especially in the major wheat-producing countries such as European Union, Russia, China, India and the United States.

### Rice

The origin of rice (*Oryza sativa*) is estimated at least 130 million years ago in Asia and has spread over the years all over the planet (Khush 1997), representing about 11% of the global cropped area. This cereal represents the primary source of food for more than one-third of the world’s population; unlike other crops, rice is consumed almost exclusively by humans (Khush 1997; Singh et al. 2018).

More than 90% of the world’s rice is grown and consumed in Asia, where it accounts for 35 to 60% of the calories consumed by 3 billion people, 60% of the worlds’ population (Khush 1997; Seck et al. 2012; Singh et al. 2018). The main producers are China, India, Indonesia and Bangladesh, with the production of 145.5; 103.5; 36.3 and 34.6 million tons, respectively (Gadal et al. 2019).

Similar to the grasses earlier mentioned, rice can also benefit from the inoculation with PGPB. Although rice is typically grown in wetland, upland cropping is very important in several countries. In wetland, rice can be associated with aerobic and anaerobic PGPB (Choudhury and Kennedy 2004). Many bacterial species have been evaluated over the years, single or associated, for growth promotion of rice, e.g. *A. lipoferum* (Watanabe and Lin 1984; Mirza et al. 2000), *A. brasilense* (de Salamone et al. 2012; Zhang et al. 2017) *Pseudomonas* spp. (Watanabe and Lin 1984; de Salamone et al. 2012; Zhang et al. 2017), *Herbaspirillum* spp. (Baldani et al. 2000; Mirza et al. 2000), *Burkholderia* spp. (Baldani et al. 2000; Tran et al. 2000; Govindarajan et al. 2007), *Bradyrhizobium* sp. (Greetatorn et al. 2019).

One of the most important studies related to inoculants for rice was carried out in Vietnam from 1999 to 2001 (Nguyen et al. 2003) and resulted in a commercial inoculant named “Biogro”. Three bacterial strains isolated from soils cropped with rice were selected and their inoculation promoted increase in grain yield compared with the non-inoculated control, reaching yields of 6.7; 6.0 and 6.2 t/ha in 1999, 2000 and 2001, respectively, when 111 kg/ha of biofertilizer were applied; the overall mean increase over the non-inoculated control was of 15% (728 kg/ha), ranging from 8.3 to 30.7%. (Nguyen et al. 2017). Similar results were obtained 1 year later in Australia, using the same mix of bacteria (Williams and Kennedy 2002).

Before 2005, the strains in “Biogro” were *Klebsiella pneumoniae* (4P), *Pseudomonas fluorescens* (1N) and *Citrobacter freundii* (3C) (Kecskes et al. 2008). From 2005 on, the inoculant was reformulated with the strains *P. fluorescens* (1N), *B. subtilis* (B9), *Bacillus amyloliquafaciens* (E19) and a soil yeast, *Candida tropicalis* (HY) (Nguyen et al. 2017). In addition to BNF, the pool of microorganisms also improved the P mobilization from soil. In field trials the new inoculant applied at a rate of 50 kg/ha promoted grain yield of 6.91 t/ha (Nguyen 2008; Nguyen et al. 2017) (Table 1). This inoculant was also efficient in rice grown on a degraded soil in the south of Vietnam (Phan and Tran 2008).

### Sugarcane

An economically important Poaceae is sugarcane. Belonging to the genus *Saccharum*, it is native from the tropical region of South and Southeast Asia (Mukherjee 1957). After many taxonomic revisions that occurred mainly during the twenty ninth century, currently the genus *Saccharum* has six species: *S. officinarum*, *S. spontaneum*, *S. robustum*, *S. sinense*, *S. barberi* e *S. edule.* Current sugarcane varieties are hybrids originating from interspecific crosses involving mainly 90% of *S. officinarum* and 10% of *S. spontaneum*. These hybrids are cited as *Saccharum* spp. (Ming et al. 2006).

America and Asia are the main sugarcane producing regions, such that in 2017 accounted for 55.7% and 37.2% of world sugarcane production, respectively (FAOSTAT 2019). The largest sugarcane producing country is Brazil, producing 758 Mt in 2017, about 41% of the world production. India, China, Thailand, Pakistan and Mexico are also important producers, contributing with 306, 104, 103, 73 e 57 Mt of sugarcane, respectively (FAOSTAT 2019).

The economic importance of this culture is related to its various purposes. Sugarcane is a raw material in the production of ethanol, biofuel widely used mainly in Brazil, in addition to the production of sugar and cane molasses, products for the food and feed industry; the vast market of products keeps its production growing continuously (Silalertruksa and Gheewala 2019).

Sugarcane is able to associate with a great diversity of diazotrophic plant growth-promoting bacteria, including species of the genera *Azospirillum* (Reis Junior et al. 2000; Tejera et al. 2005), *Azotobacter* (Tejera et al. 2005), *Burkholderia* (Perin et al. 2006; Antonio et al. 2016; Silva et al. 2016; Leite et al. 2018a, b), *Herbaspirillum* (Baldani et al. 1996; Reis Junior et al. 2000), *Pantoeae* (Taulé et al. 2012; Fischer et al. 2012, Silva et al. 2016), and the species *Gluconacetobacter diazotrophicus* (basonym *Acetobacter diazotrophicus*) (Cavalcante and Döbereiner 1988; Munõz-Rojas and Caballero-Mellado 2003; Restrepo et al. 2017), among others.

After the isolation and description of sugarcane-associated diazotrophic bacteria, and in view of the observed benefits of bacterial/plant association for other cultures, research has been intensified in Brazil. Dos Santos et al. (2018) observed the effects of inoculating a mix of diazotrophic bacteria (*G. diazotrophicus* PAL5T, *Herbaspirillum rubrisubalbicans* HCC10, *Herbaspirillum seropedicae* HRC54, *Nitrospirillum amazonense* CBAmC and *Paraburkholderia tropica* PPe4T) on sugarcane growth. After 15 days of planting, a 50% increase in dry mass of inoculated roots was observed.

The same group of bacteria was used in hydroponic sugarcane cultivation for 59 days under different concentrations of N. Two varieties of sugarcane were used: RB867515 (adapted to low fertility soils) and IACSP95-5000 (adapted to medium to high fertility soils). The authors reported that the two sugarcane varieties, when inoculated with the bacterial mix, presented different results regarding the activity of enzymes related to the assimilation of N. Under low N concentration, nitrate reductase activity was increased in RB867515 by 26% in the shoots, and by 48% in the roots, while glutamine synthetase activity was 21% higher than the control. For the IACSP95-5000 under low N concentration, nitrate reductase activity decreased by 62% in roots, and glutamine synthetase activity was increased by 16% (Dos Santos et al. 2019). This information corroborates with Schultz et al. (2017), who analyzed yield parameters in two field sites and with two sugarcane varieties (RB867515 and RB72454) inoculated or the same bacterial mix. For variety RB867515 the inoculation promoted increases in stem yield by 22.3 Mg ha^−1^ in the first site and 38.0 Mg ha^−1^ in the second site compared to the control. The variety RB72454 showed increases of 16.7 and 37.5 Mg ha^−1^, respectively.

Optimum yield results via inoculation with the same bacterial mix suggest reduced N-fertilizer application. Pereira et al. (2018) consider that inoculation coupled with the application of a low dose of N (50 kg N ha^−1^) can raise productivity with economy. In 2019 the first commercial inoculant for the sugarcane was released in Brazil, carrying *Nitrospirillum amazonense* strain.

### Pastures with grasses and legumes

Estimates are that the global pasture area covers 26% of the ice-free land surface, but in many of these places, the pastures are degraded and insufficient to provide nutrients to the animals, demanding new areas (Steinfeld et al. 2006; Fonte et al. 2014). The major problem in increasing pasture areas is that they often occur in detriment of forests, leading to deforestation, decrease in biodiversity and other environmental damages (Steinfeld et al. 2006; Don et al. 2011).

In order to improve the development of grasses in degraded pastures, the use of PGPB is once again a viable strategy. The idea is to increase soil fertility, yield and nutritional quality of grasses, decreasing the pressures on native forests (Monk et al. 2009; Campos et al. 2012; Hungria et al. 2016).

Grasslands in Brazil are estimated in 180 million ha, of which over 60 million ha are classified as degraded (LAPIG 2018), with *Brachiaria* (= *Urochloa*) representing the main component (Hungria et al. 2016). Strains Ab-V5 and Ab-V6 of *A. brasilense* have been evaluated as inoculants for *Urochloa* spp. in different sites of Brazil and the combination with N-fertilizer (40 kg ha of N) increased biomass production by 15% and of protein by 25% in comparison to the control receiving only N-fertilizer (Hungria et al. 2016). Other studies confirmed the good performance of these strains of *A. brasilense* with brachiarias (Bulegon et al. 2016; Guimarães et al. 2016; Leite et al. 2018a, b), and also with another important pasture in Brazil, panicum *Panicum maximum*, = *Megathyrsus maximus*) (Leite et al. 2019). In addition to *A. brasilense*, positive results were reported for brachiaria inoculated with *Bacillus* sp. isolated from the rhizosphere of *Urochloa brizantha* (Araujo et al. 2012).

In New Zealand, Monk et al. (2009) isolated bacteria capable of colonizing the roots of tall fescue (*Festuca arundinacea*) grasses with promising characteristics for pastures. The isolated bacteria were studied in vitro and selected for their plant-growth promotion properties, such as the production of auxins, siderophores and P solubilization.

In Colombia, *Pennisetum clandestinum* (kikuyo) was inoculated with two PGPB strains of *Stenotrophomonas* sp. and *Pseudomonas* sp. able to synthesize indole compounds, to fix nitrogen and to solubilize phosphate in vitro. Under greenhouse conditions, significant increases in the biomass and root dry weight were observed in comparison to the non-inoculated control.

Pastures with legumes are also spread all over the world, and *Trifolium* spp., *Arachis pintoi*, *Medicago sativa* L., *Stylosanthes* spp. are important examples. Dozens of studies have been performed with PGPB with those legumes. *Trifolium repens* and *Trifolium pratense* are two clovers species broadly used in pastures in Uruguay. To ensure good development there is a recommendation, since 1967, of inoculation of both clovers with *R. leguminosarum* sv. *trifolii* strain U204, a commercial inoculant strain introduced from the USA (Tartaglia et al. 2019).

Alfalfa (*Medicago sativa* L.) is present in pastures in temperate and subtropical, and arid and semi-arid areas. Buntić et al. (2019) developed a liquid-formulated inoculant containing *Sinorhizobium* (= *Ensifer*) *meliloti* strain L3Si allowing better shelf life, pre-inoculation and performance in alfalfa, as there were no liquid inoculants available with this strain. Shoot N content of plants originated from seeds pre-inoculated 1 month before sowing ranged from 3.72 to 4.19%, whereas the control with N-fertilizer had 4.03%; the highest SDW value was of 27.12 mg/plant in the inoculated plants, higher than the control with N-fertilizer (20.20 mg/plant), indicating a high effectiveness of the liquid formulation (Buntić et al. 2019).

Interest in increasing alfalfa production has also growing in Saudi Arabia. Daur et al. (2018) isolated, identified and exploited the PGPR potential of 17 bacterial isolates belonging to the genus *Bacillus*, *Acinetobacter* and *Enterobacter* from the Saudi Arabia desert and evaluated their effects on alfalfa yield. The strains were single inoculated in alfalfa seeds and sown in the fields under desert conditions. All strains improved plant relative water content, chlorophyll (a and b), carotenoids, N, P and K contents, plant height, leaf-to-stem ratio and fresh and dry weight in comparison to the non-inoculated control. However, one major consideration in this and in several other studies is the need of regulation to avoid potentially pathogenic strains in microbial inoculants, such as *Acinetobacter*, *Enterobacter* and even some species of *Bacillus*.

In Brazil, forage peanuts (*A. pintoi*) and *Stylosanthes* spp. are the most commonly used legumes in pastures. For *A. pintoi*, two *Bradyrhizobium* spp. strains are used in commercial inoculants, SEMIA 6439 (= MGAP 13) and SEMIA 6440 (= NC 230). In a field experiment that resulted in the selection of these two strains, they increased shoot dry weight, in comparison to the non-inoculated controls, without and with N-fertilizer, by 63 and 47%, respectively (Purcino et al. 2003). More recently, estimates of BNF in *A. pintoi* under field conditions were up to 65% of the total N in plants in the spring period (Carvalho et al. 2019).

Despite the widespread use of *Stylosanthes* spp. in Brazil, there are still few studies about the diversity and symbiotic efficiency of nitrogen-fixing bacteria associated to this plant. Two strains have been used in commercial inoculants, *B. japonicum* SEMIA 6155 (= BR 502) and SEMIA 6154 (= BR 446); recently, SEMIA 6154 was recognized as the type strain of a new species, *Bradyrhizobium stylosanthis* (Delamuta et al. 2016). da Chaves et al. (2016) reported that two bacterial species isolated from *Stylosanthes* (strains ERR 1178 and ERR 942 of *Bradyrhizobium* spp.) in savanna areas in Roraima, Brazil, increased the shoot biomass and N of *Stylosanthes capitata* cv. Lavradeiro under greenhouse conditions.

Australia has a long-time tradition in selecting strains and inoculating forage legumes, with emphasis on *Trifolium* spp. (Brockwell et al. 1982; Collins et al. 2002; Yates et al. 2005). More recently, in the inland areas of central Queensland, *Leucaena* has been sown and provided excellent results as forage in animal production (Buck et al. 2019); however, the inoculation of this legume is still little studied in the country.

### Vegetables

Vegetables can highly benefit from several PGPB, but this market niche is still not well explored. Taken as an example, tomato (*Solanum lycopersicum* L.) takes part in the diet of million people, consumed in salads, as ingredient of hot dishes and with great application in the industry as raw material in the manufacture of many products, mainly sauces (Subramanian 2016). Due to its versatility, tomatoes are one of the most produced vegetables worldwide. China accounts for one-quarter of world’s tomato production, followed by India and the USA (Heuvelink 2018).

Tomatoes may respond to inoculation with *Azospirillum* (Alfonso et al. 2005; Mangmang et al. 2015a; Lima et al. 2018). In Colombia, inoculation with *A. brasilense* resulted in better seedling growth, plant nutritional status, and yield 11% higher than the non-inoculated control (Alfonso et al. 2005) (Table 1).

In India, PGPB of the genera *Bacillus* and *Azotobacter* were isolated from the rhizosphere of tomatoes and tested as inoculants for this crop (Prashar et al. 2014). Previous reports from Cuba show that inoculation of tomatoes seeds with *Azotobacter chroococcum* increased plant dry weight (Puertas and Gonzales 1999). In Brazil, positive effects of inoculation of two tomatoes cultivars with *Bacillus amyloliquefaciens* subsp. *plantarum* FZB42 have also been reported (Szilagyi-Zecchin et al. 2015), increasing shoot growth, chlorophyll a, b and total, and favoring the synthesis of indole compounds and siderophores.

Several other vegetables have been reported as responsive to microbial inoculants, including lettuce (*Lactuca sativa*) (Flores-Félix et al. 2013; Mangmang et al. 2014; Fasciglione et al. 2015), carrot (*Daucus carota* L.) (Flores-Félix et al. 2013; Clemente et al. 2016) and cucumber (*Cucumis sativus* L.) (Mangmang et al. 2015b). The increasing demands of the population on organic products may also stimulate the use of microbial inoculants for the production of vegetables.

## Some of the actual threats for the use of microbial inoculants

Attention should be paid to some threats that appear from the increased scientific and commercial interest on microbial inoculants. Several studies are reporting plant-growth promoting benefits in studies with bacteria that may be harmful to plants, animals and humans. Analyzing these studies, there is no doubt that several strains of *Enterobacter* spp., of the *Burkholderia cepacia* complex, *Pseudomonas aeruginosa*, among others, can be isolated from soils and have the capacity of promoting plant growth (e.g. Adesemoye et al. 2008; Daur et al. 2018; Jung et al. 2018; Rojas-Rojas et al. 2019; Roychowdhury et al. 2019). However, they cannot be used as inoculants. Therefore, before proceeding with studies to verify the plant performance with such isolates, priority should be given to determine their taxonomic position.

In relation to agronomic practices, the compatibility with agrochemicals used for seeds treatments, with an emphasis on pesticides represents a major limitation to the survival of bacteria (e.g. Campo et al. 2009), and the problem has increased with the use of pre-inoculated seeds stored for long periods in contact with pesticides (Hungria and Mendes 2015). Priority should be given to the search for compatible agrochemicals and cell protectors (Hungria et al. 2005), or alternative technologies of application, such as the application of inoculants in-furrow to avoid the direct contact with the products used for seed treatment (Campo et al. 2010).

Amazingly, information about the benefits of microorganisms on plant growth is leading some farmers to the production of their own microbial inoculants and products for biological control. It is not difficult to perceive the threat that such practice can result to the agriculture. Production of microbial inoculants require specific requirements not easily followed even under specialized conditions (Hungria et al. 2005). Therefore, plant, human and animal pathogens have been found as predominant microorganisms in farmers´ products (Valicente et al. 2018; Hungria and Nogueira 2019) and may jeopardize the benefits of high-quality products.

## Perspectives for the future

Research on inoculants and inoculation with rhizobia and legumes raised great interest from researchers and companies in the 1970s. In the following decades, although several reports of benefits of new PGPB and the advances achieved at the inoculant industry, modest interest from research and industry has been observed. However, nowadays, increased demand for food, interest in sustainable agriculture and increasing reports on pests and pathogens resistance to agrochemicals are exponentially raising the global interest on microbial inoculants. Based on the information presented in this brief review, it is possible to perceive the increased number of studies that have been carried out about the development of new inoculants (Santos et al. 2017; Gundi et al. 2018), identification of new strains, and new inoculation methods, e.g. Zvinavashe el al. (2019), who developed a protein-based biomaterial capable of encapsulating and protecting rhizobacteria inoculated into seeds even after sowing, improving the effects of inoculation. According to information from the Web of Science database, between 2015 and 2019, 68 papers (excluding revisions) were published using the keywords “inoculant” or “biofertilizer” followed by “production” or “development”. Therefore, it is expected that in the following years innovation will be presented, encompassing both microorganisms and technologies. China currently leads the number of registered patents related to inoculation, more than 800, and India already has more than 100 inoculant industries (Fig. 2). It is expected that these numbers will also increase in other countries.

One challenge to the development of new inoculants relies on the increasing concerns about climate changes. The expected increases in temperature and dry periods in the next years will have major impacts on agriculture. According to Ramirez-Villegas and Thornton (2015), in tropical areas, maize and rice yields may decrease by 5–10% and 2–5%, respectively, for each degree of temperature increase. Climate changes will decrease the available areas for cultivation. It is therefore mandatory to search for microbial inoculants more effective under stressful conditions; on the other hand, microbial inoculants can also help to mitigate the effects of climate changes and other related abiotic stresses, such as salinity (e.g. Cerezini et al. 2016; Fukami et al. 2018b; Leite et al. 2018a, b). With increased availability of high-quality products, in addition to commitments from the governments towards more sustainable agricultural systems, the use of microbial inoculants is expected to dramatically increase in the following years.

## Data Availability

All data and materials cited in the manuscript are freely available for the scientific community.
